# Proteomic study of the inhibitory effects of tannic acid on MRSA biofilm

**DOI:** 10.3389/fphar.2024.1413669

**Published:** 2024-12-18

**Authors:** Yang Miao, Wang Shuang, Qu Qianwei, Liu Xin, Peng Wei, Yang Hai, Zhou Yonghui, Yu Xinbo

**Affiliations:** ^1^ School of Basic Medicine, Guizhou University of Traditional Chinese Medicine, Guiyang, Guizhou, China; ^2^ College of Veterinary Medicine, Northeast Agricultural University, Harbin, Heilongjiang, China; ^3^ Department of Pathology, The First Affiliated Hospital of Guizhou University of Traditional Chinese Medicine, Guiyang, Guizhou, China

**Keywords:** tannic acid, methicillin-resistant *Staphylococcus aureus*, biofilm, proteomics, inhibition

## Abstract

**Introduction:**

The mechanism of tannic acid (TA) intervention on methicillin-resistant *Staphylococcus aureus* (MRSA, USA 300) biofilm formation was explored using proteomics.

**Methods:**

The minimum inhibitory concentration (MIC) of TA against the MRSA standard strain USA 300 was determined by two-fold serial dilution of the microbroth. The effects of TA were studied using crystal violet staining. The morphology of TA-treated USA 300 cells was observed by scanning electron microscopy and confocal laser scanning microscopy. Differentially expressed proteins (DEPs) were screened using proteomic and biological information analyses, and their transcriptional levels were verified using real-time quantitative polymerase chain reaction.

**Results:**

The MIC of TA was 0.625 mg/mL, whereas 1/2 MIC (0.3125 mg/mL) of TA significantly inhibited biofilm formation without affecting the bacterial growth (*p* < 0.01) and prevented the formation of a complete three-dimensional biofilm structure. Using 1/2 MIC of TA, 208 DEPs were identified, of which 127 were upregulated and 81 were downregulated. The transcriptional levels of the genes corresponding to five randomly selected DEPs (*glnA, ribD, clpB, gap,* and *lukE*) were consistent with the proteomics data (*p* < 0.05). Bioinformatic analysis showed that the changes in the MRSA strains after TA intervention primarily involved pyrimidine and purine metabolisms, arginine biosynthesis, and the citric acid cycle.

**Conclusion:**

TA exerts an antibacterial effect on MRSA and can be used as a potential candidate for the development of anti-biofilm drugs, thereby laying a foundation for the treatment of MRSA biofilm-induced infections.

## 1 Introduction

An estimated 7.7 million deaths are attributed to bacterial infections annually, of which 4.95 million deaths are associated with drug-resistant pathogens, and 1.27 million deaths are caused by bacterial pathogens resistant to available antibiotics (Okeke et al., 2024). Drug-resistant bacterial infections are increasingly becoming a serious global health challenge. Several Gram-positive and Gram-negative bacterial strains, including *Pseudomonas aeruginosa* and methicillin-resistant *Staphylococcus aureus* (MRSA), have been identified as resistant pathogens or “superbugs” (Omeershffudin and Kumar, 2023). MRSA is a zoonotic pathogen that causes serious nosocomial infections and deaths worldwide (Otto, 2013), including skin infections, pneumonia, and sepsis. Owing to the failure of conventional antibiotic therapies, the emergence of MRSA strains has become a major clinical challenge (Sato et al., 2017; Khanal et al., 2022). Biofilm formation has been known to exacerbate antibiotic resistance (Gloag et al., 2020). A biofilm is a cluster of bacterial cells wrapped in an extracellular matrix (comprising extracellular polysaccharides, DNA, proteins, etc.) and attached to a living or inanimate surface. Under such conditions, the bacteria can evade host immunity (Zanditenas and Ankri, 2024) and increase drug resistance by more than 1,000 times. Therefore, elucidating the mechanisms of bacterial biofilm resistance and developing anti-biofilm drugs are important strategies to overcome bacterial resistance.

Tannic acid (TA) is the main bioactive compound in *Galla chinensis* and grape seeds (Chen et al., 2023a; Wang et al., 2023b); it is a polyphenolic compound that is widely found in natural medicines and can combine with proteins or alkaloids to form precipitates (Ren et al., 2021). The physiochemical properties of TA include a molecular weight of 1,701.2 g/mol, weak acidic properties, and strong astringent taste. TA has several medicinal benefits, such as anticancer, antioxidant, anti-inflammatory, and neuroprotective effects; TA can interact with biopolymers and macromolecules by crosslinking through its hydroxy and carboxy groups, making it a promising pharmaceutical candidate (Youness et al., 2021). The toxicity of TA has been confirmed previously, which has been shown to enhances hepatic function at specific concentrations. Furthermore, even when injured by acetaminophen (APAP), the hepatocytes have been known to revert to their preinjury state after TA supplementation (Lin and Hou, 2023). The pretreatment of hepatocytes with TA has been shown to alleviate subsequent damage (Lin and Hou, 2023), and studies have further demonstrated that TA treatment alleviates hepatotoxicity in APAP-induced mice, ameliorates hepatic dysfunction, and mitigates histopathological changes (Zhang et al., 2017). TA is applicable to not only viruses but also a range of bacteria, including *P. aeruginosa* and *S. aureus*, which are some of the primary factors of human infectious diseases globally. TA affects enzymatic activities owing to the presence of its phenolic hydroxyl groups (Che Lah and Kamaruzaman, 2024); phenolic compounds often play crucial roles in determining the effectiveness of antimicrobial activity and are believed to be mediated by several mechanisms, including reduced bacterial metabolism and induction of efflux pumps. TA disrupts the cell walls of microorganisms by passing through the internal membrane and disrupting the cellular metabolic reactions (Che Lah and Kamaruzaman, 2024). The efflux pump is an advanced defense system against antimicrobials; tannins have been shown to reduce the induction of efflux pumps and indirectly increase the concentrations of drugs inside the microorganisms (Che Lah and Kamaruzaman, 2024).

Previous studies have reported the antibacterial mechanisms of TA against *S. aureus* through proteomics and transcriptomics (Wang et al., 2023a). Furthermore, TA has been shown to be an effective candidate for biofilm eradication. Rendeková et al. (2015) showed that *Cotinus coggygria* Scop, which is rich in tannins and flavonoids, is a prospective topical antibacterial agent with anti-biofilm activity. Studies have shown that bacterial cellulose (BC)-based composites along with TA and magnesium chloride (BC-TA-Mg) have the potential to be used as wound dressings to combat biofilms in chronic wounds (Zhang et al., 2020; He et al., 2021). Another study has shown that tannins may directly bind to the cell-wall peptidoglycans and interfere with their integrity to achieve antimicrobial function; they may also inhibit the formation of biofilms in MRSA. Nevertheless, further research is needed to determine the mechanisms underlying the actions of tannins (Dong et al., 2018). The mechanism by which TA inhibits MRSA biofilm formation remains unclear and requires further investigation. Therefore, the present study aimed to identify and analyze the differentially expressed proteins (DEPs) during TA inhibition of MRSA biofilms through proteomics analysis. This study is expected to lay a reliable foundation for elucidating the mechanisms underlying TA disruption of MRSA biofilms and provides ideas for the development of drugs that inhibit MRSA biofilms.

## 2 Materials and methods

### 2.1 Chemicals and reagents

TA was purchased from Shanghai Yuanye Biological Company Ltd. (CAS#1401-55-4, Shanghai, China). Vancomycin was purchased from MedChemExpress, Ltd. (Cat#HY-17362/CS-0908, CAS:1404-9309, America). Crystal violet, methanol, ethanol, glacial acetic acid, absolute ethanol, glutaraldehyde, tert-butyl alcohol, phosphate-buffered saline (PBS), and other reagents were purchased from Tianjin KOMIO Chemical Reagents Company, Ltd. (Tianjin, China). Tryptic soy broth (TSB) and tryptic soy agar (TSA) were purchased from Haibo Biotechnology Ltd. (HB4114, Qingdao, China). Real-time quantitative polymerase chain reaction (RT-qPCR) kits and other related molecular biology reagents and consumables were purchased from Takara Bio (Shanghai, China). The proteomics-related reagents were provided by Hangzhou Lianchuan Biological Company (Zhejiang, China).

### 2.2 Bacterial strains

*S. aureus* USA 300, *S. aureus* ATCC 43300, and *Escherichia coli* CCTCC AB2012124 strains were provided by Professor Yanhua Li (Northeast Agricultural University, Harbin, Heilongjiang, China). *S. aureus* strains were grown in TSB medium (HB4114, Qingdao, China), and *E. coli* strains were grown in lysogeny broth medium (HB0128, Qingdao, China). All bacteria were incubated at 37°C in a constant-temperature incubator at a rotational speed of 180–200 rpm. For the biofilm assays, *S. aureus* was grown in TSB with 0.5% glucose (Zheng et al., 2024) at 37°C under static incubation.

### 2.3 Primer design

Randomly selected differential protein RT-qPCR primers were designed using Premier 5.0, and the primers were synthesized by Shanghai Sangon Biotechnology Company Ltd. (Table 1).

**TABLE 1:** Oligonucleotides used in this work.

Gene	Primer	Sequence (5′-3′)	PCR products
*16SrRNA*	Forward	AGGTCGTTATTGTTATG	136bp
	Reverse	GAGAGTTGAAGTTGGTC	
*glnA*	Forward	ATACACACGAGGATTT	220bp
	Reverse	AAGAGGTTTAGAAGGG	
*clpB*	Forward	ATA​CGT​GCT​AAT​GGT​GTT​T	117bp
	Reverse	TGA​AGT​TTC​TGA​TGA​TGC​T	
*ribD*	Forward	GTGTTTGATTGGTTGTT	107bp
	Reverse	ATTGTCTAAGTCTGGGA	
*gap*	Forward	TCGATGTGAGCTTGTGC	149bp
	Reverse	GAAGGTGGATTCCGTGT	
*lukE*	Forward	TTTGTTTTTAGGAAGGT	123bp
	Reverse	TCAGATGTGAAGGGTAG	

### 2.4 Minimum inhibitory concentration (MIC) and minimum bactericidal concentration (MBC)

Single colonies of *S. aureus* USA 300, *S. aureus* ATCC 43300, and *E. coli* CCTCC AB2012124 were selected and cultured in a sterile medium at 37°C until the logarithmic phase. The bacterial suspension was diluted to 1 × 10^5^ CFU/mL in the sterile medium. Thereafter, 100 μL of the bacterial suspension was added to each well of a 96-well microtiter plate. The MICs for the three strains were determined by two-fold serial microbroth dilutions (Alahyaribeik and Nazarpour, 2024). Next, 100 μL of a TA stock solution (40 mg/mL) was added to the first well and diluted to 12 wells, and vancomycin was added similarly to determine the MIC of TA. As a positive control, the initial vancomycin concentration was set to 250 μg/mL. Subsequently, 100 μL of the sterile medium was added to the vehicle control group, and the samples were cultured at 37°C overnight. The MIC was defined as the minimum concentration at which no visible bacterial growth was observed in the 96-well plate incubated at 37°C for 24 h. To determine the MBC, a suspension of *S. aureus* USA 300 used for MIC testing was inoculated onto TSA plates, and each assay was performed at least thrice.

### 2.5 Bacterial growth curve assays

Based on the MIC results, *E. coli* was not sensitive to TA or vancomycin; therefore, we did not determine the growth curve of *E. coli* under the actions of TA and vancomycin. Single colonies of *S. aureus* USA 300 and *S. aureus* ATCC 43300 were selected and cultured in sterile TSB medium at 37°C until the optical density (OD) at 600 nm was approximately 0.6. A bacterial suspension (5 mL) was added to each test tube, and MIC, 1/2 MIC, 1/4 MIC, and 1/8 MIC of TA were added to these tubes for each concentration; test tubes inoculated with the same concentrations of the strains without TA were used as the control group and cultured at 200 rpm in a constant-temperature shaker at 37°C. The bacterial growth curve assay of vancomycin was determined in the same manner as that for TA. The OD 600_nm_ value was measured at 1, 2, 3, 4, and 14 h after culturing, and the experiments were repeated thrice at each time point. The growth curve was then drawn using the culture time as the abscissa and measured OD 600 _nm_ value as the ordinate. All experiments were performed at least thrice.

### 2.6 Crystal violet staining assays

*S. aureus* USA 300 single colonies were cultured in sterile TSB medium at 37°C until the logarithmic phase. The bacterial suspension was then diluted to 1 × 10^5^ CFU/mL using sterile TSB medium and 100 μL of the bacterial suspension was added to each well of a 96-well microtiter plate. Subsequently, 1/2 MIC, 1/4 MIC, and 1/8 MIC of TA were added, with six replicate wells for each concentration. The six wells containing only the bacterial suspension were used as the control group, and the six wells containing only the TSB medium were used as the blank group. The crystal violet staining assay for vancomycin was determined similarly to that for TA. After incubation at 37°C for 24 h, the excess medium was removed, and the adherent biofilms were washed twice with sterile PBS. The biofilms in the wells were then fixed with 200 mL of methanol for 15 min, air-dried after removing the supernatant, and stained with 200 μL of 0.1% (w/v) crystal violet solution at room temperature for 5 min. After removing the stain, the wells were washed twice with sterile PBS. Subsequently, 200 μL of 33% (v/v) acetic acid was added to each well to dissolve the stain (Liu et al., 2022). After shaking the samples at room temperature (approximately 25°C) for 30 min, the OD was measured at 570 nm using a microplate reader (Thermo Fisher, Multiskan FC). All experiments were performed at least thrice.

### 2.7 Scanning electron microscopy (SEM) and confocal laser scanning microscopy (CLSM)

*S. aureus* USA 300 was cultured and diluted to 1 × 10^5^ CFU/mL, and 2 mL of the bacterial suspension was added to each well of a 6-well microtiter plate containing sterile glass coverslips. Then, 1/2 MIC, 1/4 MIC, and 1/8 MIC of TA were added, and the drug-free bacterial liquid well was used as the control group while the bacterial liquid well with ½ MIC vancomycin was used as the positive control group; the samples were maintained at a constant temperature of 37°C for 24 h. The coverslips were washed with sterilized PBS to remove floating bacteria; fixed with 2.5% glutaraldehyde followed by refrigeration at 4°C for 1 h; washed twice with PBS (10 min each); dehydrated with 50%, 70%, and 90% ethanol once (15 min); and dehydrated with 100% ethanol twice. The samples were then washed with 100% ethanol and tert-butanol in a 1:1 ratio (15 min). After washing once with pure tert-butanol (15 min) again, the samples were freeze-dried for 4 h. A thick metal film was placed on the surface of the sample under vacuum conditions and biofilm formation was observed using SEM (HITACHI, model SU8010, Japan). All experiments were performed at least three times.

As described above for the sample treatments for SEM, coverslips were placed over a 6-well plate to allow growth of the bacterial biofilm, and the wells were washed with PBS solution before being lightly tapped with tissue paper to maximize drainage. Staining was performed using SYTO 9 and propidium iodide (PI, Invitrogen, United States). A dye solution (20 μL) was then added to each well and incubated for 15 min. The plate was cleaned with Milli-Q water and carefully drained to remove excess water. The coverslips were carefully removed from the wells and placed on slides, and sealing oil (BacLight) was applied. The formation of fluorescent biofilms was observed under 710 nm CLSM (Leica, model TCS SP8, Germany), and the images were recorded for confirmation.

### 2.8 Proteomics assay

*S. aureus* USA 300 single colonies were selected and cultured in sterile TSB medium at 37°C until the OD 600 _nm_ value was approximately 0.6. Then, 1/2 MIC of TA (0.3125 mg/mL) was inoculated into 5 mL of sterile TSB liquid medium in a ratio of 1:100. Control test tubes were inoculated with the same concentrations of MRSA without TA and cultured at 200 rpm in a constant-temperature shaker at 37°C. After centrifugation at 4°C and 5,000×*g* for 10 min, the supernatant was discarded, bacterial precipitate was washed with PBS, and centrifugation was repeated twice. Next, protein extraction, tandem mass tag (TMT) labeling, LC-MS/MS analysis, and database searches were performed. The operating procedures of these experiments were as described previously by Chen et al. (2023b). Briefly, the bacterial samples were sonicated three times on ice using a high-intensity ultrasonic processor (Scientz) in lysis buffer (8 M urea, 1% protease inhibitor cocktail); the cell lysis solution was centrifuged at 12,000×*g* for 1 min at 4°C, and the supernatant was collected. The protein concentration of each sample was measured using a BCA protein assay kit (Cat:PC0020, Solarbio, China). Dithiothreitol, iodoacetamide, and trypsin were then added to the protein solution. After trypsin digestion, the peptides were dissolved in tetraethylammonium bromide, and the labeled reagents were dissolved in acetonitrile before mixing with the peptides. The peptides were then classified using high-pH reverse-phase high-performance liquid chromatography (HPLC). The peptides were separated using HPLC and analyzed using mass spectrometry. Secondary mass spectrometry data were retrieved using MaxQuant (v l.5.2.8), and the proteins quantified by TMT were analyzed to screen for significant differences. The TMT protein quantitative test was performed by Hangzhou Lianchuan Biological Company (Zhejiang, China). All experiments were performed at least three times.

Bioinformatics analysis was performed on the obtained data using Mascot 2.2 and Proteome Discoverer 1.4 software. For quantitative analysis, the relative quantitative value of each sample was calculated using the signal strength of the labeled reagent in the mass spectrometer, and the relative protein expression in the experimental group was compared to that in the control group. The combined method of multiple repeated experiments was used to calculate the ratio of the average values of multiple repeated experiments of the experimental to control groups. In the comparison group, the DEPs were selected according to the relative quantitative value >1.3 times or <1/1.3 and *p* < 0.05 by *t*-test. The relative quantitative value >1.3 was significantly upregulated and that <1/1.3 was significantly downregulated when using a false discovery rate of <0.01. We investigated MRSA in the UniProt database containing 29,413 sequences. Functional annotation and biological pathway analysis of the identified proteins were performed using the gene ontology (GO) and Kyoto encyclopedia of genes and genomes (KEGG) databases.

### 2.9 RT-qPCR for validating proteomics results

The *S. aureus* USA 300 single colonies were selected and cultured in sterile TSB medium at 37°C until the OD 600 _nm_ value was approximately 0.6. Then, 1/2 MIC of TA (0.3125 mg/mL) was inoculated into 5 mL of sterile TSB liquid medium in the ratio of 1:100. Test tubes inoculated with the same concentrations of MRSA without TA were used as the control group and cultured at 200 rpm in a constant-temperature shaker at 37°C. After centrifugation at 4°C and 5,000×*g* for 10 min, the supernatant was discarded, bacterial precipitate was washed with PBS, and centrifugation was repeated twice. Total RNA was extracted using an RNA extraction kit (RNAiso Plus; Code No. 9108, TaKaRa) and reverse-transcribed into cDNA using a qPCR special reverse transcription kit (PrimeScript™ RT reagent Kit with gDNA Eraser; Code No. RR047A, TaKaRa). Primers were synthesized using the sequences shown in Table 1, and 16 rRNAs were used as internal references for qPCR detection. This study was conducted using a Thermal Cycler Dice Real-Time System III (Code No. TP950) as follows: 40 cycles at 95°C for 10 min, 95°C for 15 s, and 60°C for 60 s. Relative quantification was performed using the 2^−ΔΔCt^ method, and all experiments were performed at least three times.

### 2.10 Statistical analyses

Data were analyzed using IBM SPSS Statistics for Windows (v.17.0, Armonk, NY, United States), and significant differences in values were evaluated using Student’s *t*-test (*p < 0.05). The values are reported as mean ± standard deviation (SD).

## 3 Results

### 3.1 TA affects the growth of *S. aureus*

**FIGURE 1:**
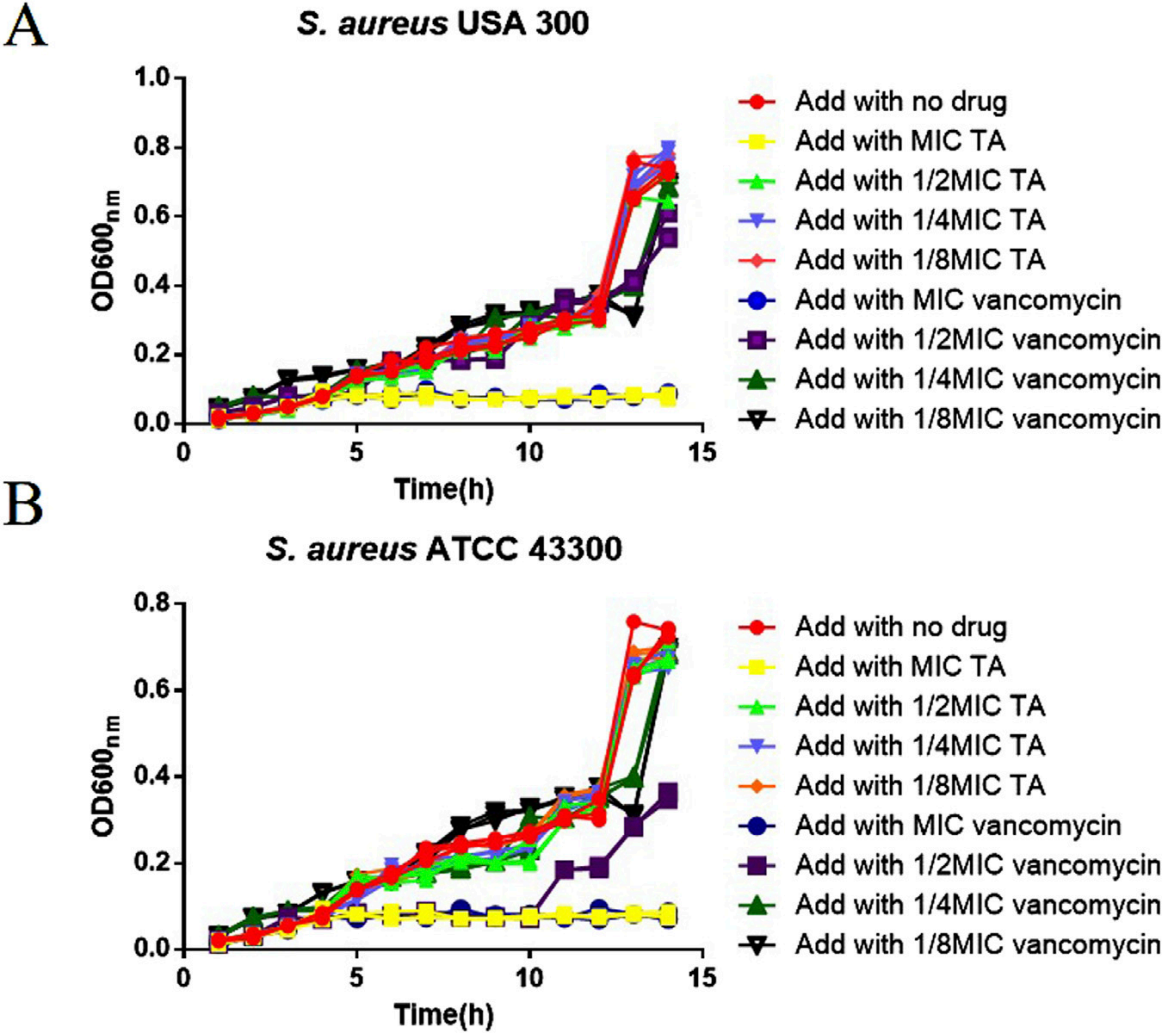
Growth curve of MRSA. **(A)** Growth curves of USA 300 after treatments with MIC, 1/2 MIC, 1/4 MIC, and 1/8 MIC of TA and vancomycin. **(B)** Growth curves of ATCC 43300 after treatments with MIC, 1/2 MIC, 1/4 MIC, 1/8 MIC of TA and vancomycin.

The MIC of TA against *S. aureus* USA 300 and *S. aureus* ATCC 43300 was 0.625 mg/mL, and the MIC of vancomycin against *S. aureus* USA 300 and *S. aureus* ATCC 43300 was 3.90625 μg/mL; *E. coli* CCTCC AB2012124 was insensitive to both TA and vancomycin (MIC results for the three strains are shown in the Supplementary Material). The MBCs of TA and vancomycin against *S. aureus* USA 300 were 1.25 mg/mL and 7.8125 μg/mL, respectively (see Supplementary Material for the results). The OD 600 _nm_ values were measured after treatments with MIC, 1/2 MIC, ¼ MIC, and 1/8 MIC of both TA and vancomycin. The results depicted in Figure 1 show that during the 1–14 h cultures of *S. aureus* in the drug treatment and control groups, the OD 600 _nm_ values increased continuously with time, the density of the bacterial solution increased, and the number of bacteria increased. After 14 h of culture, the OD 600 _nm_ value did not vary significantly (*p* > 0.05). We observed that 1/2 MIC, ¼ MIC, and 1/8 MIC of TA had no effects on the growth curve of *S. aureus* (*p* > 0.05), whereas the MIC of TA as well as 1/2 MIC, 1/4 MIC, and 1/8 MIC of vancomycin affected the growth curve of *S. aureus* (*p* < 0.05). The 1/2 MIC of TA had no effect on the growth curve of *S. aureus* and could be used to analyze the effects of TA on *S. aureus* biofilms as well as proteomics.

### 3.2 TA affects the biofilm of *S. aureus*

**FIGURE 2:**
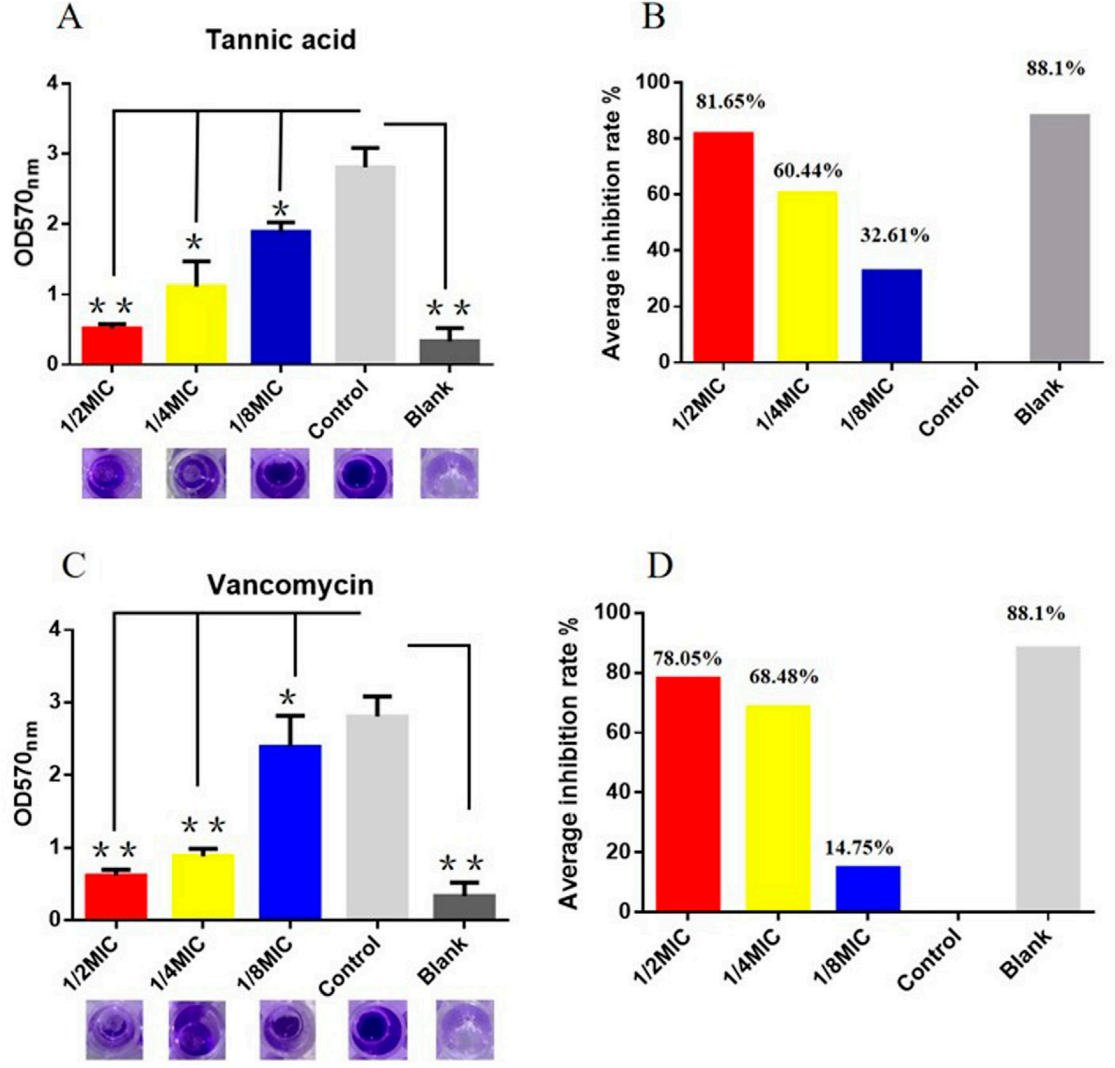
Effects of TA and vancomycin on the number of *S. aureus* USA 300 biofilms based on crystal violet staining (*P < 0.05; **P < 0.01; same as below). **(A)** OD 570 _nm_ values of *S. aureus* USA 300 biofilms after treatments with 1/2 MIC, 1/4 MIC, and 1/8 MIC of TA. **(B)** Inhibition rates of *S. aureus* USA 300 biofilms after treatments with 1/2 MIC, 1/4 MIC, and 1/8 MIC of TA. **(C)** OD 570 _nm_ values of *S. aureus* USA 300 biofilms after treatments with 1/2 MIC, 1/4 MIC, and 1/8 MIC of vancomycin. **(D)** Inhibition rates of *S. aureus* USA 300 biofilms after treatments with 1/2 MIC, 1/4 MIC, and 1/8 MIC of vancomycin.

**FIGURE 3:**
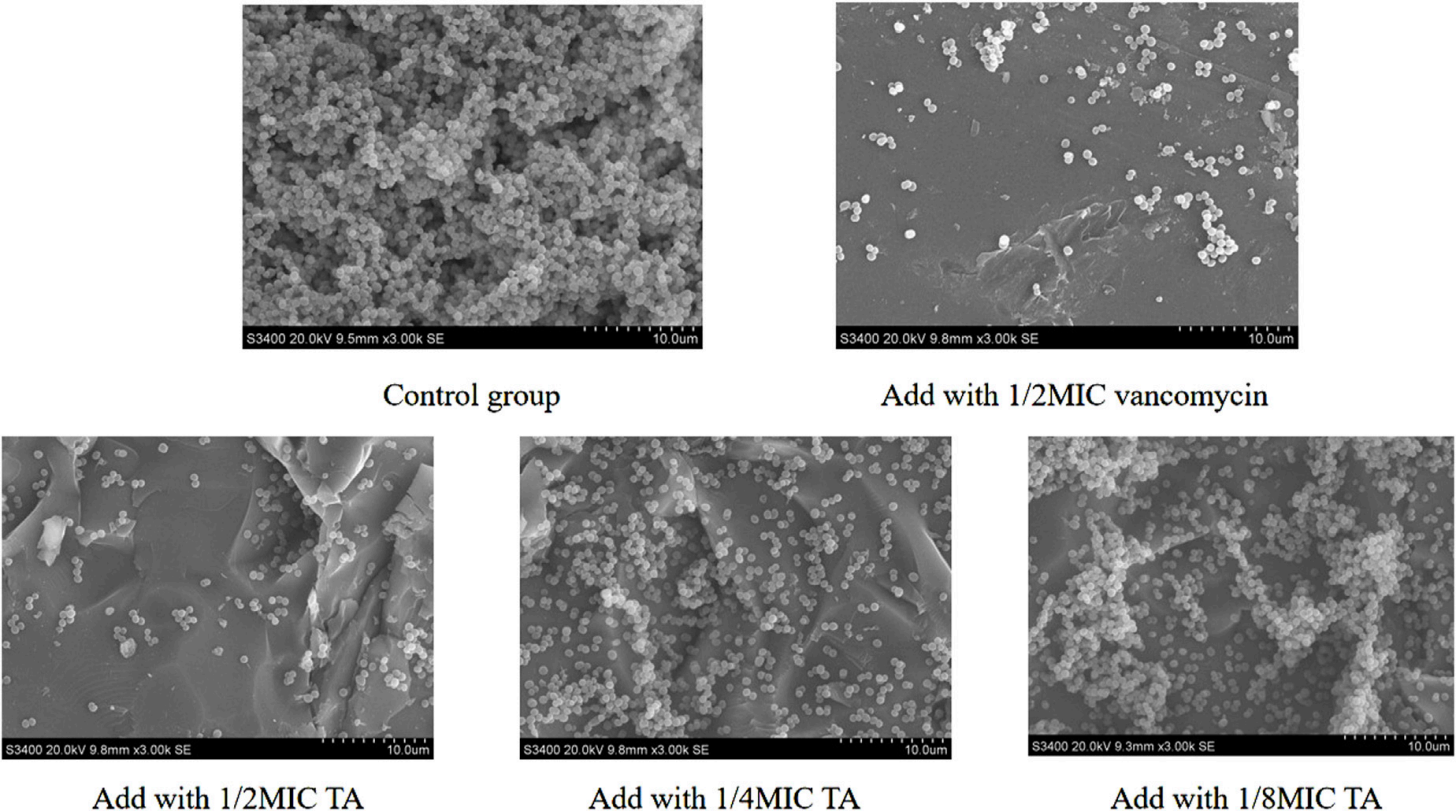
Effects of 1/2 MIC,1/4 MIC, and 1/8 MIC of TA and 1/2 MIC of vancomycin on the morphologies of *S. aureus* USA 300 biofilms using scanning electron microscopy.

**FIGURE 4:**
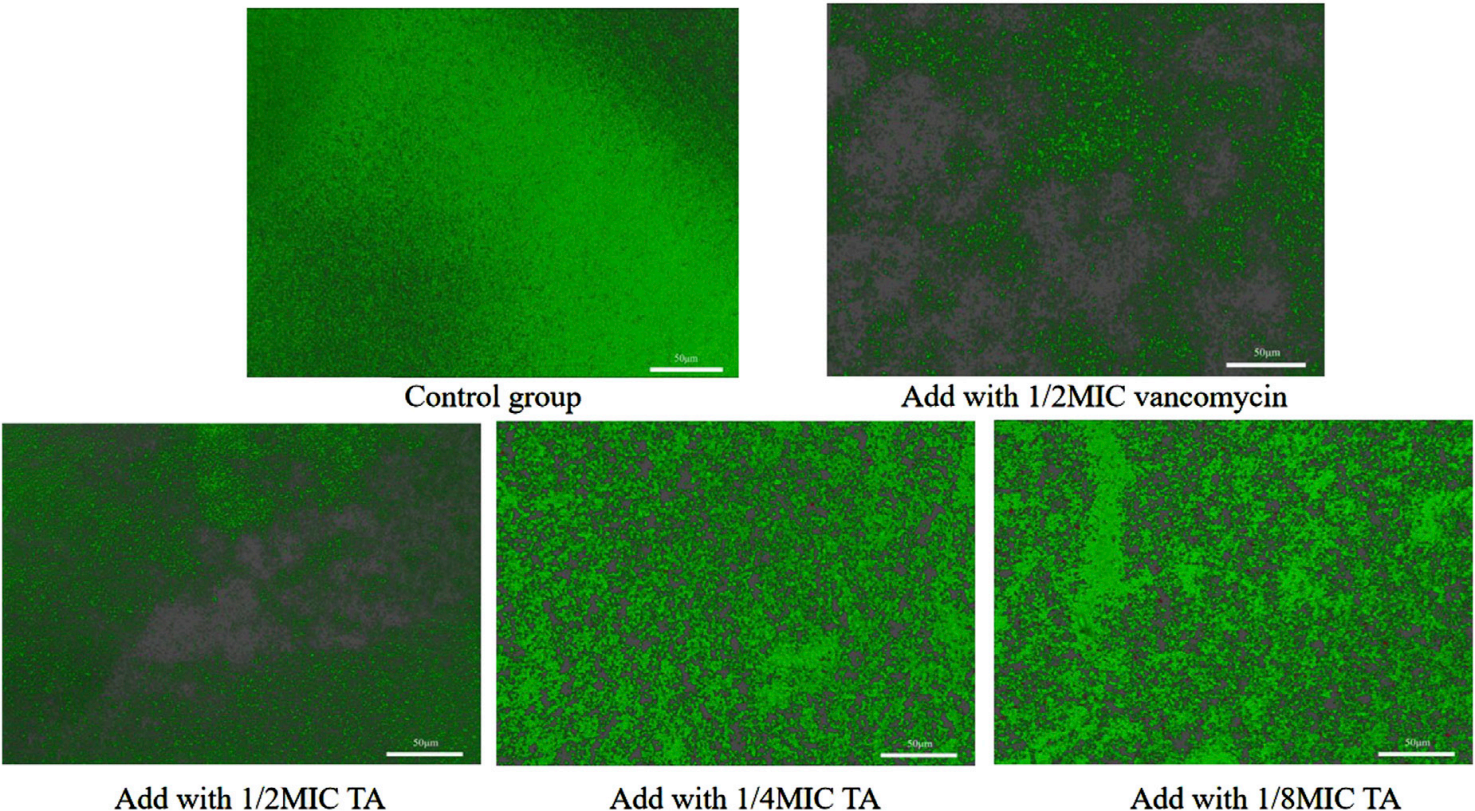
Effects of 1/2 MIC, 1/4 MIC, and 1/8 MIC of TA and 1/2 MIC of vancomycin on the biofilm integrities of *S. aureus* USA 300 observed under confocal laser scanning microscopy.

We next studied the effects of TA and vancomycin on the number and morphology of *S. aureus* USA 300 biofilms using crystal violet staining and SEM. Compared with the control group, the OD 570 _nm_ value of 1/2 MIC of TA was significantly lower (*p* < 0.01; Figure 2A) and inhibition rate was 81.65% (Figure 2B). At 1/4 and 1/8 MICs of TA, the formation of *S. aureus* USA 300 biofilms were significantly lower than those in the control group (*p* < 0.05; Figure 2A), with inhibition rates of 60.44% and 32.61%, respectively (Figure 2B). Compared with the control group, the OD 570 _nm_ values of 1/2 and 1/4 MICs of vancomycin were significantly lower (*p* < 0.01; Figure 2C), and the inhibition rates were 78.05% and 68.48%, respectively (Figure 2D). At 1/8 MIC, the formation of *S. aureus* USA 300 biofilm was significantly lower than that of the control group (*p* < 0.05; Figure 2C), with an inhibition rate of 14.75% (Figure 2D). We observed that *S. aureus* USA 300 was closely arranged, had adhered to the surface of the coverslip, and had morphological differences with the free bacteria (Figure 3). Large areas of bacterial aggregates form mature biofilms. As shown in Figure 3, only a small number of bacteria adhered to the surfaces of the coverslips under intervention with 1/2 MIC, 1/4 MIC, and 1/8 MIC of TA as well as 1/2 MIC of vancomycin, with incomplete three-dimensional structures of these biofilms. To further investigate the effects of TA on the bacterial biofilms, CLSM was used to assess the integrities of the bacterial biofilms. We observed that the control group almost completely emitted green light (Figure 4), with intact cell membrane and concentrated green fluorescence. As shown in Figure 4, under intervention with 1/2 MIC, 1/4 MIC, and 1/8 MIC of TA as well as 1/2 MIC of vancomycin, only small amounts of green fluorescence were observed, indicating that sub-MIC amounts of TA caused damage to the biofilms.

### 3.3 Proteomic profiling of *S. aureus* USA 300 treated with TA

**FIGURE 5:**
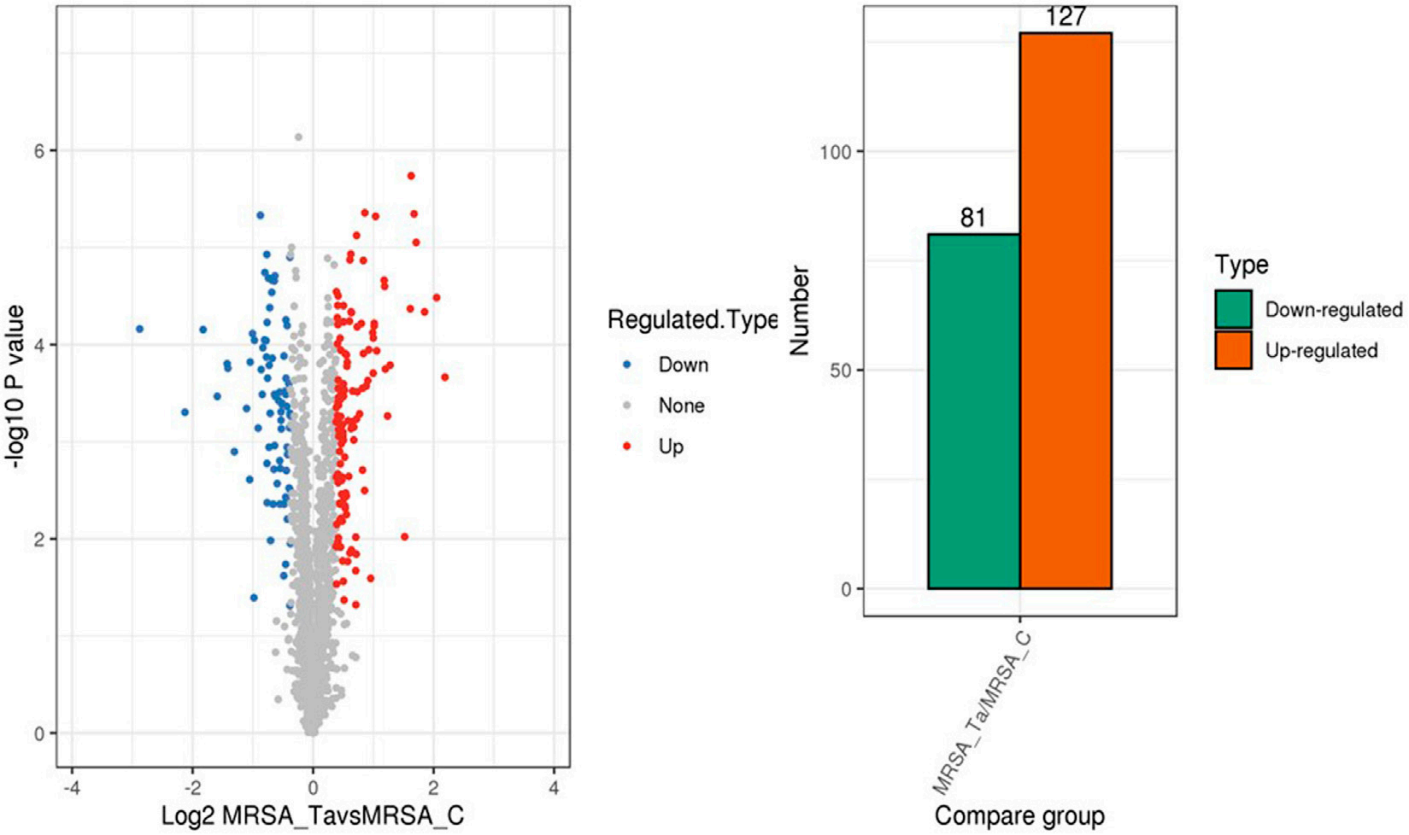
Differentially expressed proteins in MRSA (USA 300) with 1/2 MIC of tannic acid.

**FIGURE 6:**
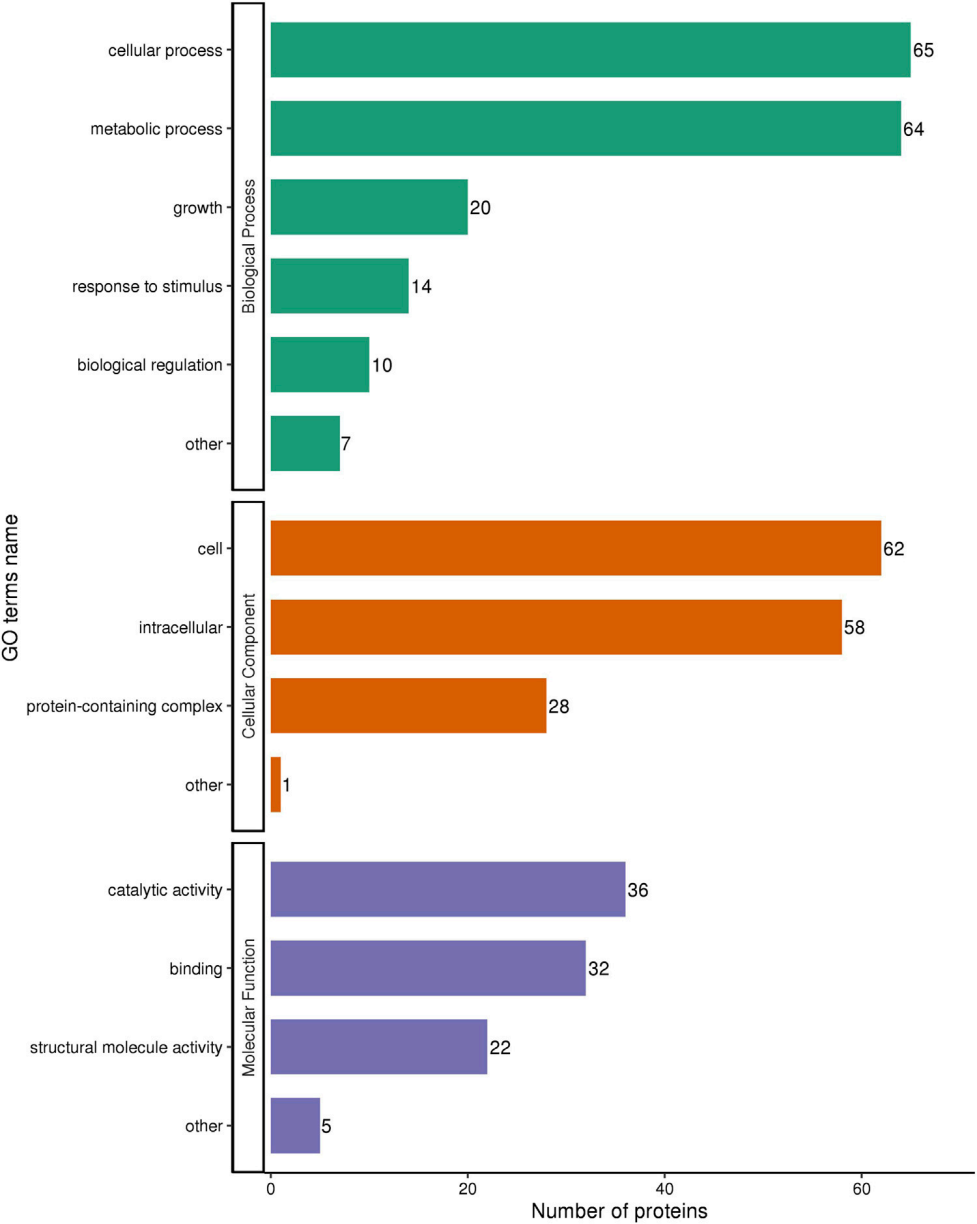
GO annotation of the differentially expressed proteins in MRSA (USA 300) with 1/2 MIC of tannic acid.

**FIGURE 7:**
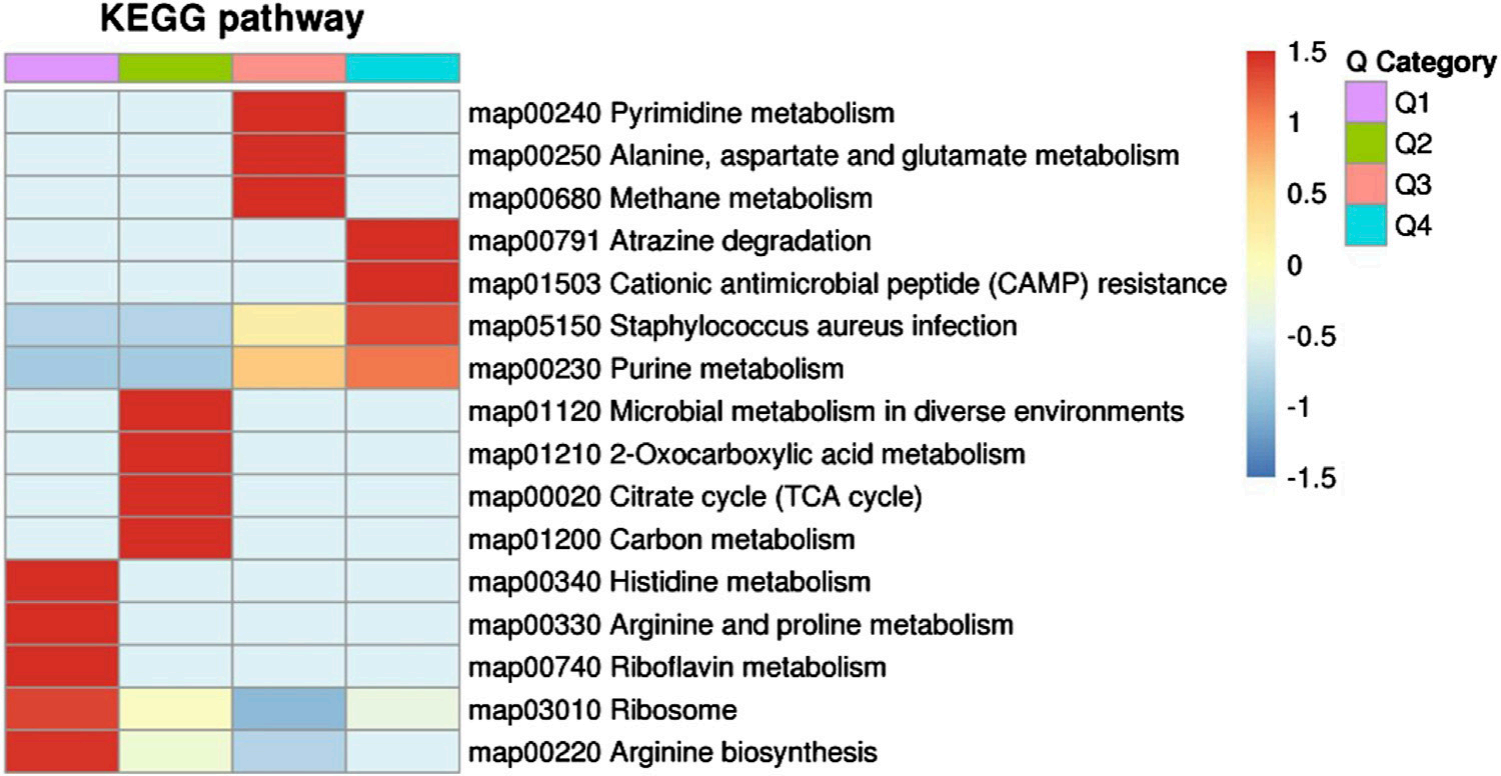
KEGG pathway clustering of the differentially expressed proteins in MRSA (USA 300) with 1/2 MIC of tannic acid.

Next, we explored the effects of 1/2 MIC of TA on the post-transcriptional landscape of *S. aureus* USA 300 using TMT-labeled proteomics. We set the cutoff criteria to a fold change >1.3 with *p*-value < 0.05 and obtained 208 DEPs, including 127 upregulated and 81 downregulated proteins (Figure 5). The GO function and KEGG pathway enrichment analyses of the differential proteins were performed to further understand the action mechanism of 1/2 MIC of TA on *S. aureus* USA 300. We found that the biological process functions were mainly related to the cellular processes, metabolic processes, and growth. The cellular components were primarily associated with cellular, intracellular, and protein-containing complexes. The molecular functions were mainly related to catalytic activity, binding, and structural molecular activity (Figure 6). KEGG analysis showed that the upregulated DEPs were enriched in related metabolic pathways, such as pyrimidine and purine metabolisms, *S. aureus* infection, and methane metabolism; the downregulated DEPs were enriched in the citric acid cycle, carbon metabolism, histidine metabolism, arginine biosynthesis, and other related metabolic pathways (Figure 7).

### 3.4 RT-qPCR for validating proteomics results

**FIGURE 8:**
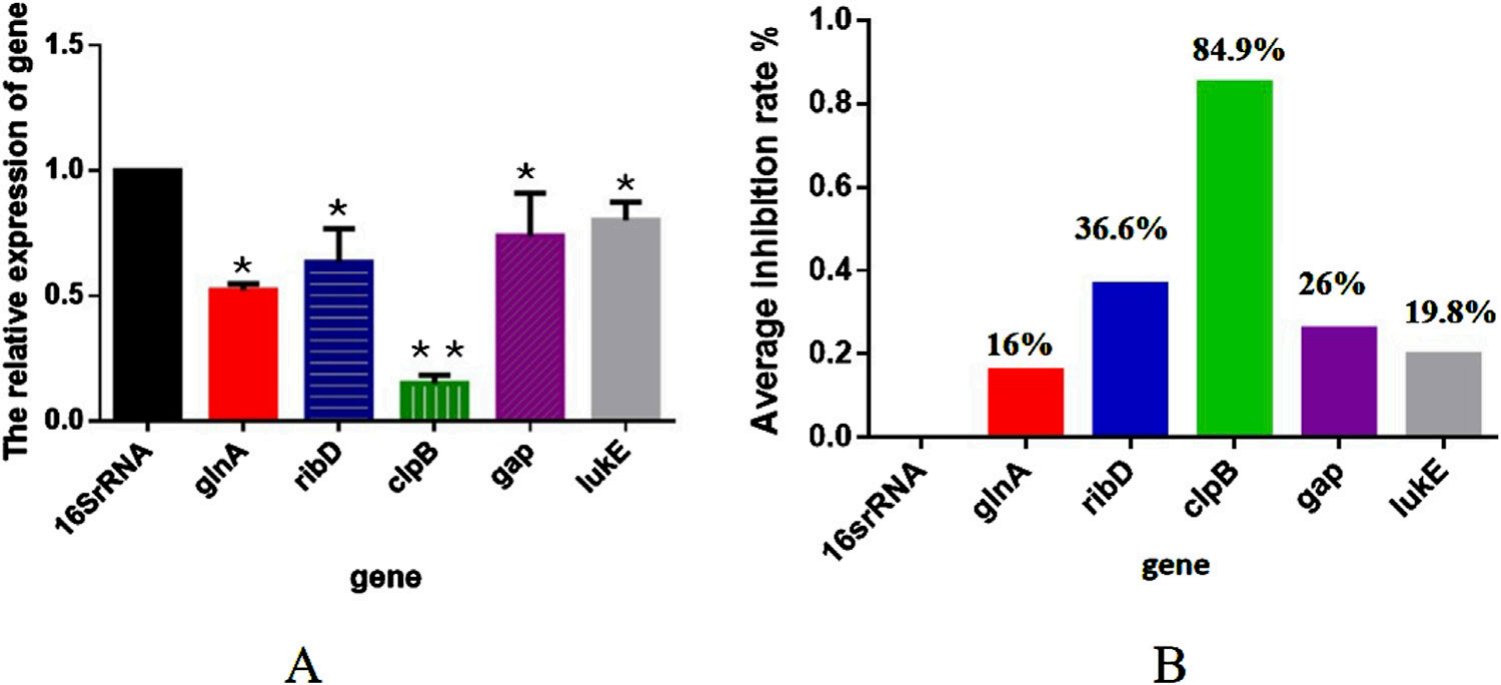
**(A)** Transcription levels of the differentially expressed proteins in MRSA (USA 300) with 1/2 MIC of tannic acid. **(B)** Inhibition rates of the transcription levels of the differentially expressed proteins in MRSA (USA 300) with 1/2 MIC of tannic acid.

To verify the proteomics results, we selected five DEPs with the potential to inhibit the proliferation of *S. aureus* and analyzed their transcription level changes using RT-qPCR. As illustrated in Figure 8, when *S. aureus* USA300 was treated with 1/2 MIC of TA, the expressions of *glnA, ribD, clpB, gap,* and *lukE* were significantly downregulated (*p* < 0.05), consistent with the proteomics results. These findings further confirmed the accuracy of the proteomics results.

## 4 Discussion

TA is a natural and process-derived phenolic compound that is a reportedly effective antagonist to viruses and bacteria (Dong et al., 2018). Our results indicate that TA may be an effective candidate for the development of novel strategies to treat MRSA infections (Dong et al., 2018). Although TA is regarded as a natural bioactive component, its application to combating clinical pathogens and bacterial resistance remains largely unexplored. TA has been reported to have antibacterial effects and to reduce drug resistance. However, its mechanism of action requires further investigation. In this study, we investigated the antibacterial activity and related mechanisms of action of TA against *S. aureus*. Based on crystal violet staining and SEM experiments, we found that 1/2 MIC, 1/4 MIC, and 1/8 MIC of TA significantly inhibited *S. aureus* biofilm formation. The concentration ranges in a dose-dependent manner, indicating that TA at sub-inhibitory concentrations may effectively prevent *S. aureus* biofilm formation. We also found that 1/2 MIC of TA had no inhibitory effect on the growth of *S. aureus*, and it was speculated that TA may affect biofilm formation through other factors such as metabolism and regulation in *S. aureus* cells.

SEM and CLSM revealed that 1/2 MIC, 1/4 MIC, and 1/8 MIC of TA effectively inhibited the formation of three-dimensional structures of MRSA biofilms. Studies (Maisetta et al., 2019) have shown that TA can inhibit the formation of biofilms of *S. aureus* and *S. epidermidis*, but its specific mechanism has not been elucidated. TA may also increase the sensitivity of bacteria to antibiotics (Qin et al., 2023). In addition, it reduces bacterial biofilm formation and colonization by inducing the expression of the immunodominant staphylococcal antigen A (IsaA). These studies have shown that TA inhibits the formation of *S. aureus* biofilm; however, the underlying mechanisms remain unclear. Therefore, it is boldly envisaged that TA could be a potential candidate for the development of new antibiotics against bacterial-associated infections.

To further elucidate the mechanism by which TA interferes with *S. aureus* biofilm formation, a TMT-labeled proteomics relative quantitative method was used to screen for DEPs in MRSA and MRSA treated with 1/2 MIC of TA. The results revealed 208 DEPs (including 127 upregulated and 81 downregulated) that were mainly involved in nitrogen and carbon metabolisms. A previous study revealed the antibacterial mechanism of TA against *S. aureus* through proteomics and transcriptomics (Wang et al., 2023a). In the present study, we focused on the protein changes associated with biofilm inhibition to identify the key proteins responsible for drug inhibition of biofilm formation and provide ideas for follow-up studies on biofilm inhibitors.

Reverse transcription RT-qPCR is the gold standard for exploring transcriptional variations; it is often the easiest and most cost-effective method of measuring gene expression and understanding complex regulatory networks (Koch et al., 2019). The use of reference genes is the most commonly accepted method of normalization (Koch et al., 2019). As shown in Figure 8, five randomly selected significantly downregulated genes, namely, *glnA*, *ribD*, *clpB*, *gap*, and *lukE*, had inhibitory effects on *S. aureus*. Biofilm formation by *S. aureus* on any surface can be facilitated by adjusting its redox status; this organism is a facultative anaerobe that shifts toward reductive conditions by enhancing nitrogen metabolism, in which glutamine synthesis plays a key role. Glutamine is synthesized by glutamine synthetase encoded by the *glnA* gene, whose sequence shows a high degree of variability in its human counterpart but is highly conserved in bacteria (Gowrisankar et al., 2021). Heat shock proteins (Hsps) play essential roles in stress responses, function as molecular chaperones to stabilize proteins, and aid in protein refolding under stressful conditions (Yang et al., 2024). ClpB (Hsp100) is a major molecular chaperone expressed in bacteria, protozoa, fungi, and plants but not in animals or humans; it can disaggregate stress-denatured proteins along with the DnaK system, thus protecting bacteria from a range of stressors, including heat, acidity, and oxidation (Yang et al., 2024). Over the past few decades, ClpB has been investigated in both Gram-negative and Gram-positive bacteria, revealing its role in stress responses and virulence (Kędzierska-Mieszkowska and Arent, 2020). *S. aureus* secretes cytolytic toxins called leukocidins to injure immune cells; among these, leukocidin (LukED) has the widest tropism in human white blood cells (Poddighe and Vangelista, 2020). *LukE/D* is reportedly one of the most important pore-forming toxin genes in *S. aureus* isolates as it lyses the host cells and promotes bacterial virulence (Zhu et al., 2022). Pathogens can either synthesize this vitamin *de novo* through the riboflavin biosynthetic pathway (RBP) or scavenge it from host tissues using flavin transport systems to ensure cell proliferation and survival (Gnanagobal et al., 2023). The RBP produces riboflavin from guanosine-5-triphosphate (GTP) and ribulose-5-phosphate using the activities of five enzymes: GTP cyclohydrolase II (RibA according to gram-negative bacterial nomenclature), 3,4-dihydroxy-2-butanone-4-phosphate (3,4-DHBP) synthase (RibB), a bifunctional pyrimidine deaminase/reductase (RibD), riboflavin synthase (RibE), and 6,7-dimethyl-8-ribityllumazine (lumazine) synthase (RibH) (Gnanagobal et al., 2023). GapDH is a conserved target for redox regulation and post-translational thiol modifications, including S-glutathionylation, across all life domains (Hillion et al., 2017). In *S. aureus*, glycolytic GapDH was recently shown to be a major target for *S*-bacillithiolation, contributing to 4% of the total Cys proteome (Hillion et al., 2017). GapDH uses the Cys active site for nucleophilic attack on the aldehyde group of glyceraldehyde-3-phosphate to catalyze its phosphorylation to 1,3-bisphosphoglycerate, thereby generating NADH (Hillion et al., 2017).

Pyrimidine metabolism, purine metabolism, histidine metabolism, arginine biosynthesis, and other nitrogen-metabolism-related pathways were also significantly enriched. Pyrimidine and purine nucleotides interact with the bacterial secondary messenger cyclic diguanylate (c-di-GMP), which is involved in the regulation of bacterial biofilm synthesis as well as degradation, motility, toxicity, cell cycle, cell differentiation, and other activities (Spangler et al., 2011). TA may affect interaction with c-di-GMP by acting on the pyrimidine and purine metabolisms, resulting in inhibition of MRSA biofilm formation. In addition, histidine metabolism plays an important role in biofilm formation by *S. xylosus* and may be the target of cefquinome to inhibit biofilm formation (Zhou et al., 2018). In the present study, TA significantly inhibited the histidine metabolic pathway, which may have affected biofilm formation. Manna et al. (2022) and Jing et al. (2022) showed that arginine biosynthesis is involved in biofilm formation by *S. aureus* and *S. mutans*. In contrast, Nishimura et al. (2022) showed that arginine inhibited the formation of *S. cerevisiae* biofilms. We found that the metabolic pathway for arginine synthesis was significantly downregulated after TA treatment. In summary, nitrogen metabolism plays an important role in the effect of TA on *S. aureus* biofilm formation.

We also found that methane metabolism, citric acid cycle, carbon metabolism, and other metabolic pathways related to carbon metabolism were significantly enriched. The citric acid cycle may regulate production of golden yellow pigments by regulating the flow of acetyl-CoA into the mevalonate pathway and then into the pigment synthesis pathway. The citric acid cycle is closely associated with bacterial pathogenicity (Bhusal et al., 2019). Proteins in the citric acid cycle pathway are reportedly downregulated in both cotrimoxazole- and amikacin-resistant *K. pneumoniae* (Shen et al., 2021), which may inhibit reactive oxygen species production in drug-resistant isolates. The proteomics results showed deficiencies of resistant *K. pneumoniae* in the central metabolic pathways (mainly citric acid cycle) compared with sensitive *K. pneumoniae*. De Backer et al. (2018) further showed that the citric acid and urea cycles affect extracellular matrix synthesis and regulate the formation of MRSA biofilm. The present study shows that TA not only inhibits methane metabolism but also inhibits the citric acid cycle and carbon metabolism pathway, indicating that TA may affect the carbon metabolism of *S. aureus*, resulting in a decrease in biofilm formation. This suggests that carbon metabolism is an important regulatory factor in TA intervention of *S. aureus* biofilm formation.

In summary, the current study shows that TA inhibits MRSA activity. Bacteriostasis was achieved mainly by inhibiting the nitrogen metabolism, carbon metabolism, and other related pathway proteins of MRSA. Although we have demonstrated a possible mechanism underlying the antibiofilm activity of TA, the determinants of such activity require further study.

## 5 Conclusion

The present study shows that naturally active tannic acid has good antibacterial activity against MRSA. Tannic acid can be used as a foundation for further research on the inhibitory mechanisms of MRSA biofilm formation and drug development.

## Data Availability

The data presented in the study are deposited in the Science data bank. Research Data at https://doi.org/10.57760/sciencedb.18488.
