# Supplementary material for: Proteomic study of the inhibitory effects of tannic acid on MRSA biofilm
Source: Front Pharmacol. 2024 Dec 18;15:1413669. doi: 10.3389/fphar.2024.1413669 (PMC11688184; doi:10.3389/fphar.2024.1413669)
Supplement: Supplementary file 1 [file DataSheet1.docx]

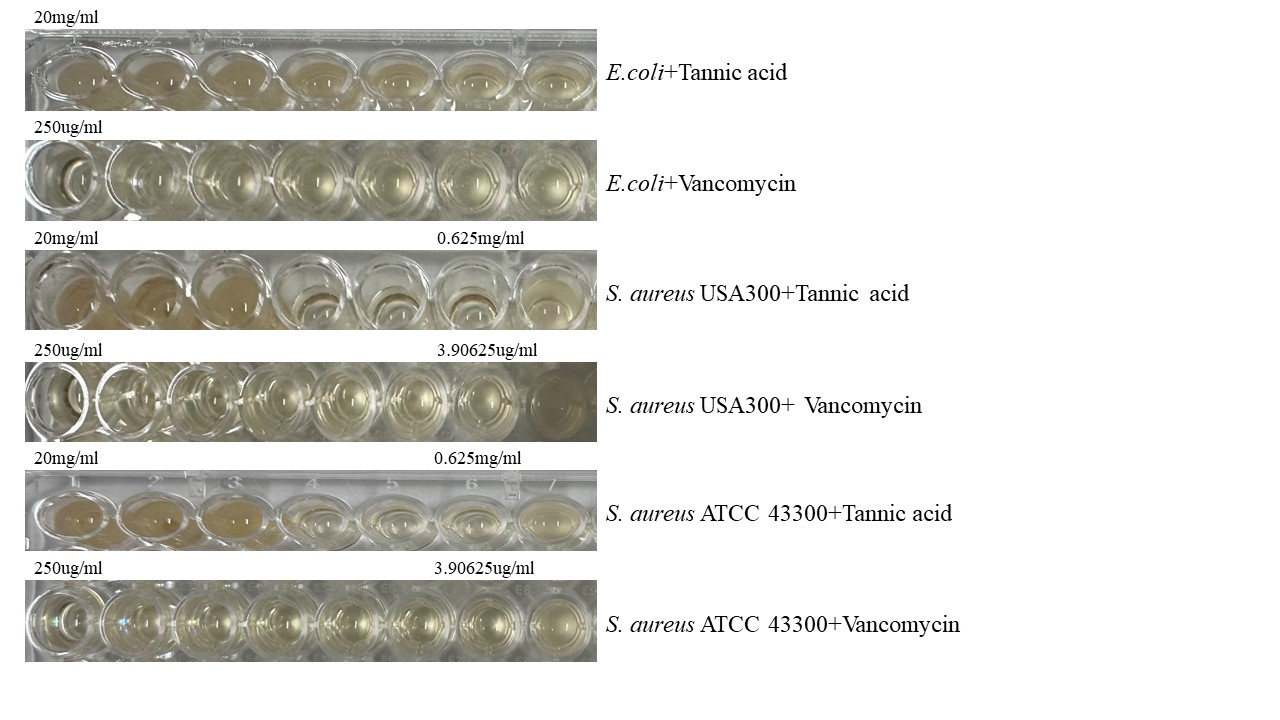


Remarks: Supplementary materials(MIC of three bacteria of *S. aureus* USA300、*S. aureus* ATCC 43300 and *E. coli*)


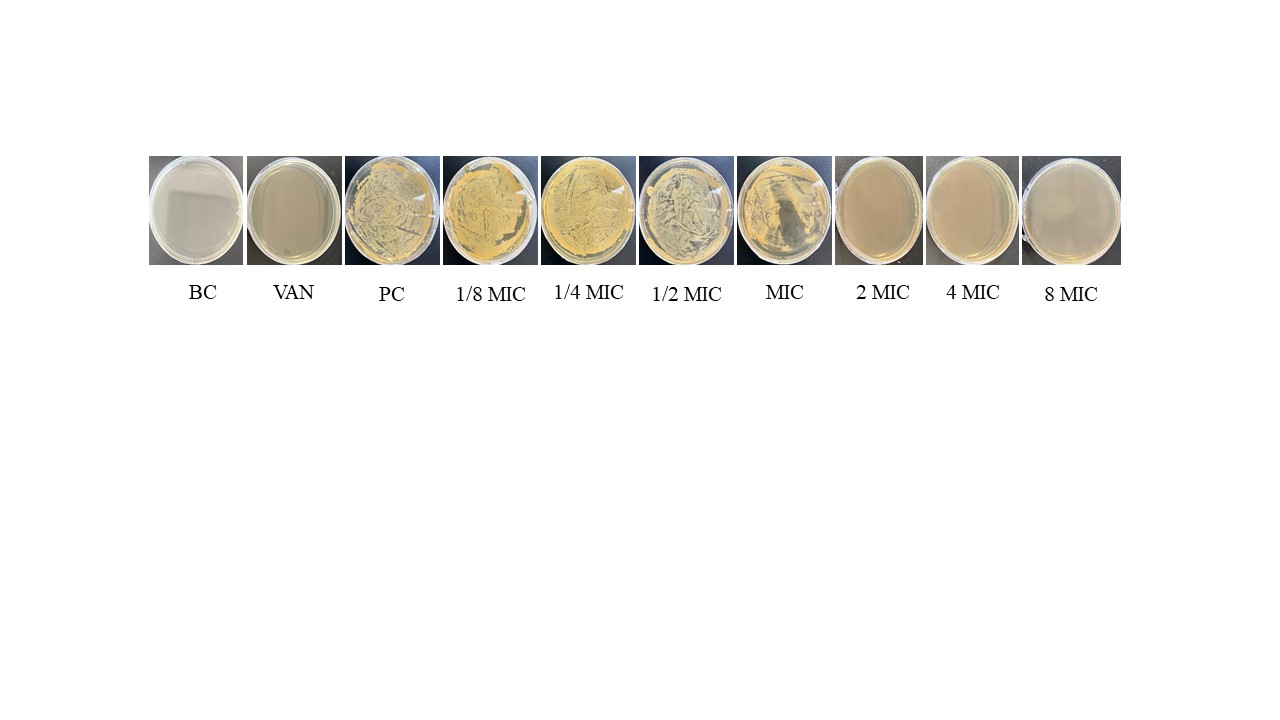


Remarks: BC=Blank control, VAN=vancomycin, PC=*S. aureus* USA300(MBC of *S. aureus* USA300)
